# MscaVPR: Multi-Scale Coordinate Attention Network for Robust Visual Place Recognition

**DOI:** 10.3390/s26103261

**Published:** 2026-05-21

**Authors:** Xiaohan Gao, Zhinong Zhong, Yongjian Tan, Ning Jing, Anran Yang, Qingren Jia

**Affiliations:** College of Electronic Science and Technology, National University of Defense Technology, Changsha 410073, China; gaoxiaohan@nudt.edu.cn (X.G.); zzn@nudt.edu.cn (Z.Z.); tanyongjiannudt@nudt.edu.cn (Y.T.); ningjing@nudt.edu.cn (N.J.); yanganran@nudt.edu.cn (A.Y.)

**Keywords:** visual place recognition, multi-scale feature aggregation, viewpoint robustness

## Abstract

Visual place recognition (VPR) aims to localize a query image by matching its visual representation against a geotagged database. One major challenge in VPR is to learn place representations that remain robust under appearance changes, viewpoint variations, and perceptual aliasing. However, existing VPR methods still show limitations in adaptive multi-scale feature fusion and viewpoint-aware training supervision, which may hinder robustness under severe viewpoint changes. In this paper, we propose MscaVPR, a VPR framework that combines multi-scale feature enhancement with azimuth-aware training. Specifically, a Multi-Scale Spatial Pyramid Attention (MSPA) module is incorporated to aggregate regional features across different spatial scales, and Coordinate Attention (CA) is used to encode positional cues for spatially refined feature learning. To further enhance viewpoint robustness, we design an azimuth-guided training strategy that selects hard positive samples with significant viewpoint discrepancies and optimizes them using an azimuth-aware auxiliary loss function.Experimental results on multiple benchmark datasets indicate that MscaVPR generally outperforms the strong baseline and demonstrates improved performance under challenging conditions. In particular, Recall@1 is improved by 2.1%, 1.9%, and 1.9% on the AmsterTime, SVOX-Night, and SVOX-Sun datasets, respectively. The results demonstrate that explicitly incorporating azimuth cues provides an effective complement to existing multi-scale and attention-based VPR methods.

## 1. Introduction

Visual place recognition (VPR) is a fundamental task in computer vision and robotics, aiming to identify a geographical location solely from the visual features of an image, without the aid of GPS information [1]. This task is typically formulated as an image retrieval problem [2], in which the goal is to find images in a database that are visually similar to a given query without requiring complex 3D reconstruction, thus enabling relatively direct and efficient localization. VPR has applications in virtual reality [3], augmented reality [4], autonomous vehicles [5], environmental exploration [6], robotic localization [7], and smart cities [8], where robust performance under varying environmental conditions is critical.

**Figure 1:**
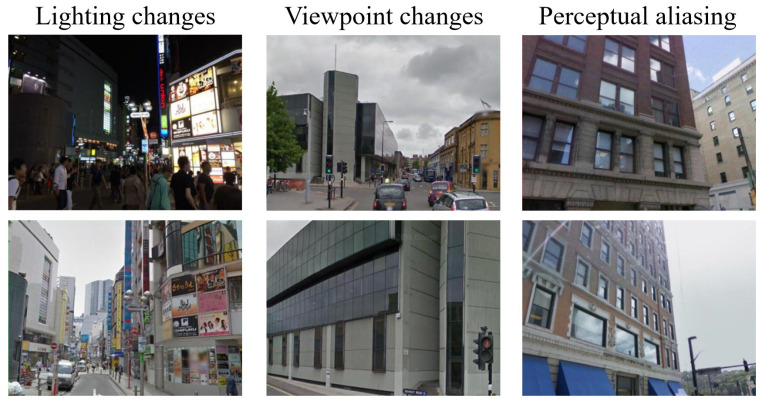
Challenging scenarios in the VPR task.

The standard image-retrieval-based VPR pipeline consists of two main stages: extracting image descriptors to construct a searchable feature database and retrieving the most similar database images for a given query according to descriptor similarity [9,10]. As a result, the quality of feature representation largely determines the final retrieval performance. However, learning robust place representations in real-world outdoor environments remains challenging as a result of to three tightly coupled factors: appearance variations caused by illumination, season, and weather changes; viewpoint variations induced by changes in camera orientation and position; and perceptual aliasing, where different places exhibit highly similar visual patterns [2]. Examples of these challenging scenarios are shown in Figure 1.

To address these challenges, recent VPR methods have increasingly relied on deep visual representations. CNN-based approaches improve robustness through hierarchical feature extraction and global aggregation, such as NetVLAD [1], and GeM [11], while later methods further enhance discriminability via regional modeling and multi-scale aggregation. More recently, Transformer-based architectures have shown a strong ability to capture long-range dependencies and contextual relationships, with attention-based methods such as TransVPR [12] and CricaVPR [13] achieving notable progress in challenging VPR scenarios.

Despite these advances, two limitations remain. First, regional cues in existing methods are often integrated in a fixed or only weakly adaptive manner, with limited explicit modeling of spatial positional information, which may hinder the full exploitation of complementary cues across spatial scales and viewpoints. Second, most methods still adopt generic metric learning objectives and conventional positive-pair construction, without explicitly leveraging viewpoint-related cues such as image azimuth. Consequently, hard positive pairs with large directional discrepancies are often insufficiently emphasized during training, weakening robustness to severe viewpoint changes. These limitations motivate us to improve viewpoint robustness from both representation learning and training supervision, where azimuth metadata provide a lightweight and practical cue for guiding viewpoint-aware optimization.

Building upon the strong CricaVPR framework [13], this paper presents MscaVPR, which improves viewpoint-robust visual place recognition through the joint design of feature enhancement and azimuth-aware training. The main contributions of this work can be summarized as follows:

(1) We enhance place representation with multi-scale and coordinate-aware feature modeling. Specifically, a Multi-Scale Spatial Pyramid Attention (MSPA) module [14] is introduced to adaptively aggregate regional features across different spatial scales, and Coordinate Attention (CA) [15] is incorporated to encode positional cues and refine spatial responses.
(2) We propose an azimuth-guided training strategy to explicitly improve robustness to viewpoint changes. By leveraging azimuth metadata, the proposed strategy preferentially selects hard positive samples with larger viewpoint discrepancies and further optimizes them through an azimuth-aware auxiliary loss function.
(3) Extensive experiments on both standard and challenging benchmarks verify the effectiveness of the proposed method and demonstrate that explicitly incorporating azimuth cues consistently improves viewpoint robustness over a strong baseline.

## 2. Related Work

### 2.1. Traditional Place Recognition with Handcrafted Features

Early VPR methods were primarily built on handcrafted local features, such as SIFT [16], SURF [17], HOG [18], and ORB [19]. These descriptors were typically combined with global encoding schemes, including Bag-of-Words (BoW) [20], Fisher Vector (FV) [21], and Vector of Locally Aggregated Descriptors (VLAD) [22], to form compact image-level representations suitable for large-scale retrieval. Such methods established the early foundation of place recognition by enabling efficient matching across large reference databases.

Despite their effectiveness in controlled environments, methods based on handcrafted features are inherently limited by their sensitivity to photometric and geometric variations. Changes in illumination, weather, season, or camera viewpoint often cause local descriptors to become unstable or ambiguous. Moreover, these methods mainly rely on bottom-up matching of local structures and therefore lack robust semantic understanding of the scene as a whole, which makes them particularly vulnerable to perceptual aliasing in environments with repetitive visual patterns. These limitations motivated the transition to data-driven deep representations.

### 2.2. CNN-Based Learned Global Representations

The emergence of deep convolutional neural networks shifted VPR from hand-engineered pipelines toward end-to-end feature learning. CNN backbones such as VGG [23] and ResNet [24] enabled hierarchical visual representations that are substantially more robust to appearance changes than handcrafted descriptors are. A major milestone in this direction was NetVLAD [1], which introduced a differentiable VLAD layer on top of convolutional features, allowing the entire retrieval pipeline to be optimized directly for place recognition. This framework demonstrated the effectiveness of jointly learning local feature extraction and global descriptor aggregation. Patch-NetVLAD [25] further improved robustness by incorporating patch-level residual aggregation, preserving local details under appearance and viewpoint changes. Subsequent CNN-based research mainly focused on more effective aggregation and training strategies. MixVPR [26] replaced residual-based aggregation with MLP-based feature mixing, enabling efficient global descriptor learning through iterative spatial and channel interaction. CosPlace [27] adopted a classification-based training scheme that avoids explicit positive–negative pair construction, greatly improving training efficiency. To enhance viewpoint robustness, GeoWarp [28] learned geometric transformations to align features across camera positions, and EigenPlaces [10] encoded viewpoint invariance into global descriptors through contrastive learning on the large-scale SF-XL dataset [27]. Despite these advances, multi-scale cues in CNN-based VPR are often integrated in an implicit or relatively fixed manner, while adaptive cross-scale fusion of hierarchical features remains limited.

### 2.3. Attention-Based and Transformer-Based VPR

Attention-based methods further advanced VPR by improving the modeling of discriminative regions and long-range contextual dependencies. Early studies showed that Vision Transformers (ViTs) were able to outperform CNN-based descriptors for image retrieval [29], highlighting the benefit of global context modeling for place recognition. TransVPR [12] used multi-level attention to enhance discriminative patches while jointly capturing local and global cues. BoQ [30] formulated global representation learning as query-based cross-attention aggregation, producing compact and discriminative descriptors with high efficiency. Moving beyond single-image reasoning, CricaVPR [13] introduced cross-image correlation awareness to exploit complementary information across multiple views of the same place, but its emphasis is primarily on inter-image relational modeling rather than explicit cross-scale self-adaptation fusion within image representations. ClusVPR [31] further improved region selection by using clustering-based weighted Transformers to emphasize representative and diverse image regions, enhancing robustness to viewpoint changes and scene complexity. More recently, EffoVPR [32] showed that pretrained vision foundation models already encode highly transferable cues for VPR, and this model leveraged internal Transformer features and attention maps within a lightweight retrieval framework to achieve strong performance under challenging appearance and viewpoint changes. However, coordinate-aware spatial reweighting is still less explored in existing attention-based VPR methods, and the preservation of directional positional information during long-range multi-scale interactions remains limited.

## 3. Methodology

### 3.1. Network Architecture

**Figure 2:**
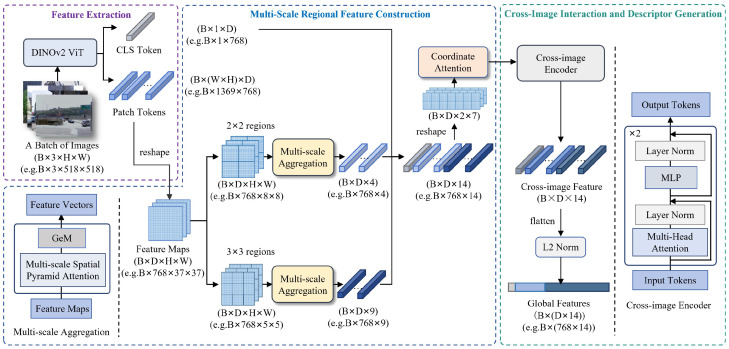
Overall architecture of MscaVPR. The feature extraction and partitioning as well as the cross-image encoder are inherited from CricaVPR, while the feature aggregation stage is enhanced in this work by integrating MSPA-based multi-scale regional aggregation and coordinate attention. For clarity, the dimensions shown in the partition branches denote the feature size of each regional input; regions within the same scale are processed independently and then pooled into regional descriptors.

Herein, to improve visual representation learning under appearance and viewpoint variations, we develop MscaVPR as an enhanced variant of CricaVPR [13]. As shown in Figure 2, the framework preserves the backbone feature extraction and cross-image correlation pipeline of CricaVPR, while strengthening the feature aggregation stage by introducing MSPA-based multi-scale regional aggregation and coordinate-aware refinement. Specifically, the Multi-Scale Spatial Pyramid Attention (MSPA) module [14] is integrated into each regional aggregation branch to enhance structural representation at different spatial scales, and Coordinate Attention (CA) [15] is employed to encode positional cues among regional descriptors before cross-image encoding.

Given an input image, DINOv2 ViT-B/14 [33] is used as the backbone to extract one CLS token and a set of patch tokens. The CLS token serves as a global representation, while the patch tokens preserve local spatial information. The patch tokens are reshaped into a 2D feature map $F\in R^{B\times D\times H\times W}$ and partitioned into 2×2 and 3×3 spatial grids, yielding 4 and 9 sub-regions, respectively. After independent MSPA processing and pooling, these sub-regions produce 13 regional descriptors of dimension *D*, which are concatenated into $R^{B\times D\times 13}$ and further combined with the CLS token to form the image-level feature sequence $R^{B\times D\times 14}$. This sequence is reshaped into a pseudo-spatial tensor $R^{B\times D\times 2\times 7}$ and refined by the CA module to preserve positional sensitivity and emphasize informative structural regions while suppressing background interference. The refined output is then reshaped back to $R^{B\times D\times 14}$ and processed by the cross-image encoder inherited from CricaVPR to model inter-image correlations. Finally, the output tokens are flattened into $R^{B\times (D\cdot 14)}$ and L2-normalized to obtain the global descriptor for image retrieval.

### 3.2. Feature Aggregation Based on Multi-Scale Spatial Pyramid Attention

**Figure 3:**
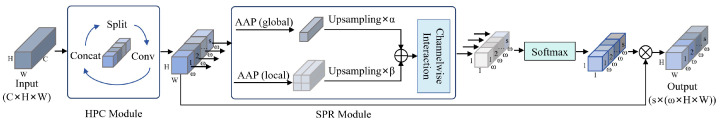
Detailed structure of the MSPA module adopted in MscaVPR. MSPA consists of Hierarchical Phantom Convolution (HPC), Spatial Pyramid Recalibration (SPR), and Softmax-based channel recalibration. In our framework, MSPA is integrated into each scale-specific regional aggregation branch to enhance the structural representation of partitioned patch feature regions before GeM pooling.

To exploit multi-scale spatial information during feature aggregation, we incorporate the Multi-Scale Spatial Pyramid Attention (MSPA) module proposed in [14] into MscaVPR. In the original work, MSPA is used to replace the 3×3 convolution in the bottleneck residual blocks of CNN backbones. In contrast, we adapt it to the regional aggregation stage of the VPR pipeline, where it refines the partitioned patch features at each spatial scale before regional pooling. The architecture of MSPA is illustrated in Figure 3.

Following Ref. [14], MSPA contains three components: Hierarchical Phantom Convolution (HPC), Spatial Pyramid Recalibration (SPR), and Softmax-based channel recalibration. The HPC module splits the input feature map along the channel dimension and processes each group through hierarchical residual-like convolutional paths, allowing spatial responses with different receptive fields to be aggregated. The SPR module combines global average pooling and local average pooling to capture complementary contextual information and incorporates lightweight pointwise convolutions to learn channel relationships. The resulting channel attention weights are normalized by Softmax to model long-range channel dependencies and are then applied to the corresponding feature subsets for recalibration.

In MscaVPR, MSPA is applied independently to the regional feature of each spatial sub-region. For both the 2×2 and 3×3 branches, the output of MSPA retains the same feature dimension as its input, so that each region can be consistently transformed into a 768-dimensional descriptor after GeM pooling. In this way, the multi-scale aggregation branch yields regional descriptors with a unified embedding space, which are then concatenated with the CLS token to form the image-level feature sequence for subsequent coordinate-aware refinement and cross-image encoding.

### 3.3. Coordinate-Aware Feature Refinement

**Figure 4:**
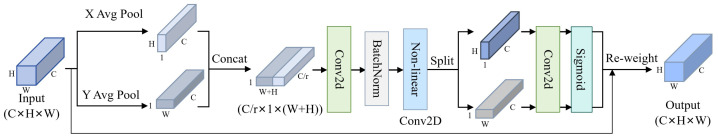
Detailed structure of the Coordinate Attention (CA) module used in MscaVPR. The concatenated token sequence is reordered into a 2×7 pseudo-spatial grid, where CA encodes long-range dependencies along horizontal and vertical directions while preserving regional positional cues. The refined features are then reshaped back into the token sequence for subsequent cross-image encoding.

After multi-scale aggregation, we apply Coordinate Attention (CA) [15] to refine the concatenated image feature sequence before cross-image encoding. CA embeds positional information into channel attention by decomposing 2D global pooling into two one-dimensional encoding processes along the horizontal and vertical directions. This design allows the module to capture long-range dependencies along one spatial direction while preserving positional cues along the other. In MscaVPR, CA is used as a lightweight coordinate-aware recalibration module for the concatenated multi-scale regional tokens. The structure of CA is shown in Figure 4.

Given the concatenated feature sequence, we first reshape it into a pseudo-spatial grid $X\in R^{C\times H\times W}$, where H=2 and W=7 in our implementation. CA aggregates features along the two spatial directions using 1D average pooling:

(1) $$z_{c}^{h}\left(h\right)=\frac{1}{W}\sum_{i=1}^{W}x_{c}\left(h,i\right)$$

(2) $$z_{c}^{w}\left(w\right)=\frac{1}{H}\sum_{j=1}^{H}x_{c}\left(j,w\right)$$

The two direction-aware descriptors are concatenated and transformed by a shared 1×1 convolution, followed by normalization and non-linear activation. The transformed feature is then split into two branches and projected to generate height-aware and width-aware attention maps, denoted as $g^{h}$ and $g^{w}$. The output of CA is computed as

(3) $$y_{c}\left(i,j\right)=x_{c}\left(i,j\right)\times g_{c}^{h}\left(i\right)\times g_{c}^{w}\left(j\right)$$

The refined feature grid is finally reshaped back into a token sequence and fed into the cross-image Transformer encoder inherited from CricaVPR. This coordinate-aware refinement preserves lightweight computation while providing position-sensitive regional representations for subsequent inter-image correlation modeling.

### 3.4. Azimuth-Guided Positive-Pair Selection

Images captured at the same geographic location may still exhibit substantial visual differences when their viewing directions differ significantly. Although such samples remain valid positives, they are generally more challenging than those with similar azimuths. To exploit this property during training, we incorporate the azimuth information provided by the northdeg field in the street-view dataset and use it to preferentially select hard positive samples.

For each image, the raw azimuth annotation is first normalized to the range [0°, 360°) using a modulo-360° operation. Given two images *i* and *j* captured at the same location, their circular azimuth difference is defined as

(4) $$d_{ij}=\min\left(\left|\phi_{i}-\phi_{j}\right|,\;360{}^{\circ}-\left|\phi_{i}-\phi_{j}\right|\right)$$

where $\phi_{i}$ and $\phi_{j}$ denote the normalized azimuth angles of samples *i* and *j*, respectively. To further account for the approximate 180° ambiguity caused by sensor noise, heading misalignment, or annotation inconsistency, we map the angular difference into the effective range of [0°, 90°] as follows:

(5) $${\tilde{d}}_{ij}=\min\left(d_{ij},180{}^{\circ}-d_{ij}\right)$$

This formulation reduces the influence of inconsistent directional annotations while preserving the relative viewpoint discrepancy between two images.

As summarized in Algorithm 1, hard positive selection is performed within each place-level image group during training. Specifically, we first randomly sample *K* images from the same geographic location, where *K* is determined by img_per_place, and denote their azimuth annotations as $\left\{\phi_{1},\phi_{2},\ldots ,\phi_{K}\right\}$. Based on the pairwise effective azimuth differences ${\tilde{d}}_{ij}$, we construct a difference matrix and consider only its upper-triangular entries to avoid duplicate pairs. All candidate pairs are then ranked by ${\tilde{d}}_{ij}$, and the top pairs M=⌊K/2⌋ with the largest azimuth differences are selected as hard positive pairs. The image indices involved in these selected pairs are merged to form the hard-positive subset. If the number of unique selected images is smaller than *K*, the remaining positions are filled by randomly sampling from the unselected images, so that a fixed number of training images are retained for each place. In our implementation, K=4, and thus, the two pairs with the largest azimuth differences are selected within each place-level group.
**Algorithm 1** Azimuth-guided hard positive sampling within a place**Require:** Place-level image set S, number of sampled images *K*
**Ensure:** A selected image subset $S_{hard}$ of size *K*
  1:Randomly sample *K* images from S to obtain $\left\{I_{1},\ldots ,I_{K}\right\}$  2:Extract azimuth annotations $\left\{\phi_{1},\ldots ,\phi_{K}\right\}$  3:Normalize azimuths by $\phi_{i}\leftarrow \phi_{i}\;\mathrm{mod}\;180{}^{\circ}$  4:**for** i=1 to *K* **do**  5:    **for** j=i+1 to *K* **do**  6:        Compute $d_{ij}\leftarrow \left|\phi_{i}-\phi_{j}\right|$  7:        Compute ${\tilde{d}}_{ij}\leftarrow \min\left(d_{ij},\;180{}^{\circ}-d_{ij}\right)$  8:    **end for**  9:**end for**10:Collect all upper-triangular pairs (i,j) and their scores ${\tilde{d}}_{ij}$11:Rank all pairs by ${\tilde{d}}_{ij}$ in descending order12:Select the top M=⌊K/2⌋ pairs13:Merge the image indices from the selected pairs into an index set I14:**if** |I|<K **then**15:    Randomly sample additional indices from {1,…,K}∖I16:    Add them to I until |I|=K17:**end if**18:Construct $S_{hard}=\left\{I_{i}|i\in I\right\}$19:**return** $S_{hard}$


**Figure 5:**
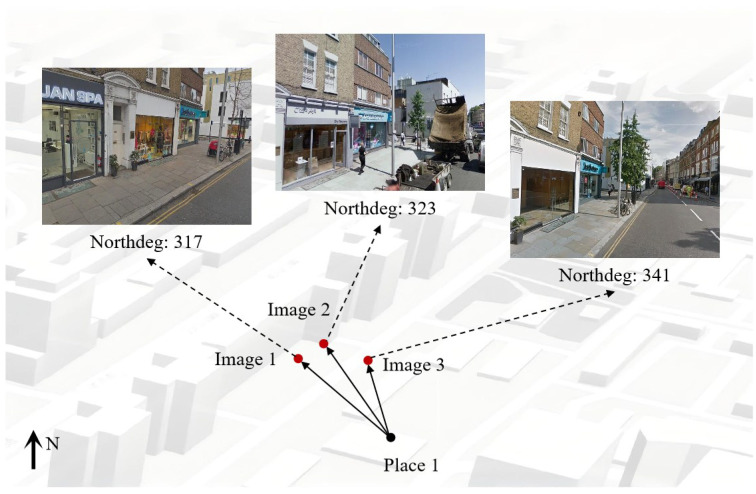
Illustration of azimuth-guided hard positive selection. Images captured at the same location but with larger viewing-direction differences are preferentially selected as harder positive samples during training.

As illustrated in Figure 5, this azimuth-guided strategy favors positive samples with larger viewpoint variation and thereby increases the difficulty of matching within places during training while remaining fully compatible with the original metric learning framework.

### 3.5. Loss Function

To optimize similarity learning while explicitly accounting for viewpoint variation, we employ a hybrid objective that combines multi-similarity (MS) loss with an azimuth-aware auxiliary term. The MS loss [34] is defined as

(6) $$L_{MS}=\frac{1}{m}\sum_{i=1}^{m}\left\{\frac{1}{\alpha }\log\left[1+\sum_{j\in P_{i}}e^{-\alpha (S_{ij}-\lambda )}\right]+\frac{1}{\beta }\log\left[1+\sum_{j\in N_{i}}e^{\beta (S_{ij}-\lambda )}\right]\right\}$$

where $P_{i}$ and $N_{i}$ denote the positive and negative sets associated with sample *i*, respectively; $S_{ij}$ is the cosine similarity between samples *i* and *j*; α and β control the weighting of positive and negative pairs; and λ is the similarity margin threshold.

To further enhance robustness to spatial viewpoint variations, we introduce an azimuth-aware regularization term and define the overall training loss as

(7) $$L_{\mathrm{total}}=L_{MS}+\gamma L_{\mathrm{ang}},$$

where γ is the weighting factor of the azimuth term. To avoid introducing an additional hard margin beyond the thresholding mechanism already embedded in $L_{MS}$, we define azimuth loss using a smooth Softplus-like penalty:

(8) $$L_{\mathrm{ang}}=\frac{1}{\left|H\right|}\sum_{(i,j)\in H}\theta_{ij}\log\left(1+e^{-S_{ij}}\right)$$

where *H* denotes the set of hard positive pairs mined during training. This formulation assigns a larger penalty to hard positive pairs with low similarity while remaining easy to optimize jointly with $L_{MS}$. The azimuth weight $\theta_{ij}$ is defined as

(9) $$\theta_{ij}=\frac{{\tilde{d}}_{ij}}{90{}^{\circ}}$$

where ${\tilde{d}}_{ij}$ is the effective azimuth difference defined in Equation (5). Thus, $\theta_{ij}\in \left[0,1\right]$, and larger values are assigned to positive pairs with greater viewpoint discrepancies.

Consequently, when an image pair depicting the same geographic location exhibits a large azimuth difference with low feature similarity, the corresponding loss value increases significantly. This encourages the model to learn feature representations that are more robust and invariant to viewpoint changes.

## 4. Experiments

### 4.1. Datasets and Evaluation Metrics

We train the proposed model on the GSV-Cities dataset [35], a large-scale benchmark for visual place recognition that contains approximately 560,000 street-view images collected from more than 67,000 geographic locations in multiple cities worldwide. Each location is associated with four to twenty images, and different locations are separated by at least 100 m. Owing to its broad geographic coverage and diverse appearance variations, GSV-Cities has been widely adopted for training VPR models.

During training, Pitts250k-val [36] is used as the validation set. For evaluation, we conduct experiments on seven VPR benchmarks, the main characteristics of which are summarized in Table 1. Pitts250k [36] consists of Google Street View images collected in downtown Pittsburgh and exhibits notable viewpoint and appearance variations. Tokyo 24/7 [37] contains query images captured at 125 locations in central Tokyo at different times of day and viewing directions, making it suitable for evaluating robustness to illumination and viewpoint changes. Eynsham [38] is composed of grayscale images from urban and rural road scenes, which increases the difficulty of place recognition owing to the absence of color cues. MSLS [39] spans more than seven years and includes urban, suburban and natural environments, thus covering substantial appearance changes over time.

In addition, we evaluate the model on three challenging datasets characterized by severe environmental variation and domain shift. AmsterTime [40] contains 1231 query-database pairs that match historical black-and-white photographs to modern RGB street-view images, posing a difficult long-term and cross-domain retrieval task. The SVOX series [28], derived from the Oxford RobotCar dataset, is designed to assess retrieval performance under extreme environmental conditions, including sunny, rainy, night, and snowy scenes. In this work, we use the SVOX-Night and SVOX-Sun subsets, which emphasize severe illumination and weather changes.

We adopt Recall@N as the evaluation metric, following previous VPR studies [13]. Recall@N measures the percentage of queries for which at least one of the top *N* retrieved database images is considered correct. For datasets with GPS annotations, a retrieval is treated as correct if the matched database image lies within 25 m of the ground-truth query location.

### 4.2. Implementation Details

All experiments are conducted in PyTorch 1.11.0 on two NVIDIA GeForce RTX 3090 GPUs. We follow the training protocol of GSV-Cities [35] and use a batch size of 72. Each batch is constructed from 72 distinct locations, with four images sampled per location. The input image resolution is 224×224. We adopt DINOv2 ViT-B/14 as the backbone network, with a token embedding dimension of 768. The final descriptor produced by our model consists of token-level global features with a size of 14×768, which are further reduced to 4096 dimensions using Principal Component Analysis (PCA) for retrieval evaluation.

We optimize the network using the Adam optimizer with an initial learning rate of $1\times 10^{-4}$. The learning rate is not fixed during training; instead, it is decayed by a factor of 0.5 every three epochs according to a step-based schedule. Early stopping is employed when Recall@5 on the validation set does not improve for three consecutive epochs. For the multi-similarity loss in Equation (5), we set α=1, β=50, and λ=0, and we adopt a margin of 0.1 for online hard sample mining, following [35]. For the azimuth-aware loss term in Equation (6), the weighting coefficient γ is determined through validation-based tuning. Specifically, we evaluate γ∈{0.1,0.3,0.5,0.7} and observe that γ=0.3 yields the most favorable and stable overall performance. Therefore, this value is used throughout the remaining experiments.

Since VPR training is affected by stochastic factors such as data sampling, batch construction, and hard mining, each experiment is repeated three times using different random seeds. We report the mean and standard deviation of all quantitative results to provide a more reliable assessment of performance.

### 4.3. Comparisons with State-of-the-Art Methods

To evaluate the effectiveness of the proposed method, we compare it with several representative state-of-the-art VPR approaches, including SFRS [41], CosPlace [27], EigenPlaces [10], CricaVPR [13], ClusVPR [31], and BoQ [30]. The quantitative results are summarized in Table 2 and Table 3. Specifically, Table 2 reports results on four commonly used benchmark datasets, while Table 3 presents performance on three more challenging datasets with larger variations in illumination, weather, and domain shift.

As shown in Table 2, MscaVPR performs competitively across the evaluated benchmark datasets. It achieves the best mean R@1 results on Pitts250k-test, Tokyo 24/7, and Eynsham, outperforming the second-best methods, CricaVPR and BoQ, by 0.6, 0.8, and 0.2 percentage points, respectively. On MSLS-val, MscaVPR remains highly competitive, with a mean R@1 only 0.1 percentage points lower than that of BoQ. Although limited, these improvements are stable across repeated runs, and the results show that the proposed method consistently maintains competitive performance on standard urban benchmarks while remaining effective on larger and more diverse datasets.

The results in Table 3 further demonstrate that MscaVPR consistently outperforms all compared methods on the three more challenging datasets. On AmsterTime, which involves substantial temporal gaps and cross-domain appearance changes, MscaVPR improves R@1 from 63.0% (CricaVPR) to 65.1%, highlighting its ability to handle long-term visual discrepancies. On SVOX-Night and SVOX-Sun, which are characterized by severe illumination, weather, and day/night variations, MscaVPR achieves R@1 scores of 90.1% and 95.1%, respectively, exceeding the second-best method, BoQ, by 1.2 and 1.5 percentage points. These results confirm that MscaVPR is effective not only on standard benchmarks but also in challenging real-world scenarios involving substantial temporal, cross-domain, and environmental variations.

**Figure 6:**
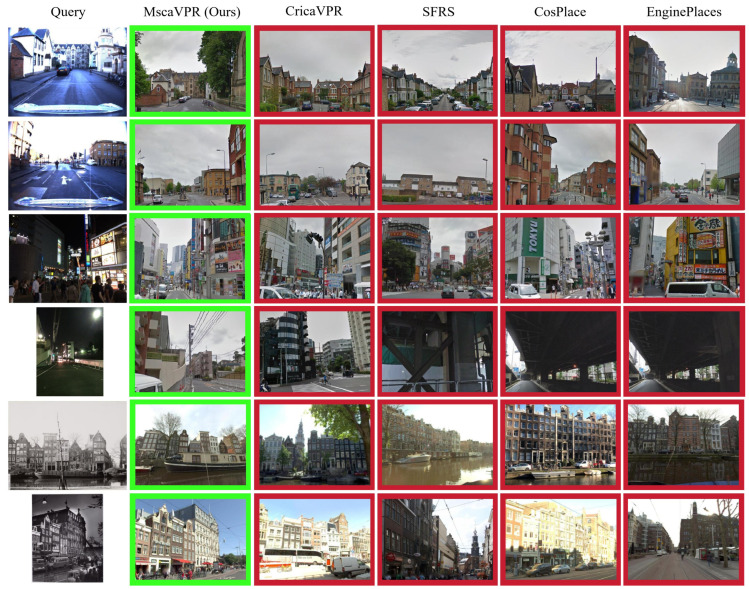
Qualitative results of our model and the SOTA methods in challenging cases. The correct results are marked with green boxes, and the incorrect results are marked with red boxes.

Figure 6 presents qualitative comparisons on several difficult examples. In these cases, the query images differ from the matched database images not only in viewpoint but also in environmental conditions. The first two examples are affected by severe overexposure, while the third and fourth correspond to nighttime scenes. Despite these challenges, our method successfully retrieves the correct matches. The last two examples further involve large temporal gaps, leading to architectural modifications and color discrepancies between historical and contemporary images. Even under these conditions, MscaVPR is still able to identify the correct locations, which is consistent with its quantitative improvements on AmsterTime.

**Figure 7:**
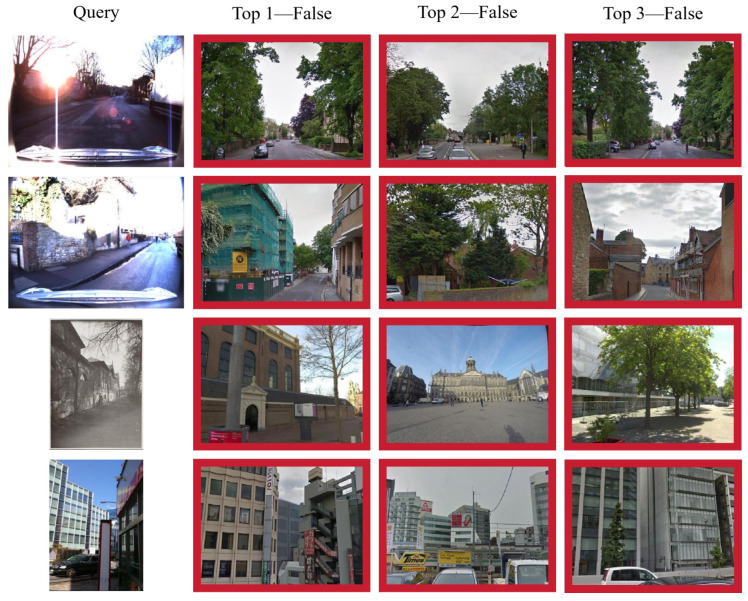
Cases of failed top 3 retrieval results with our model. The incorrect results are marked with red boxes.

Figure 7 shows representative failure cases in which none of the top three retrieved results are correct. These queries are affected by extreme imaging conditions, such as severe glare, strong backlighting, and heavy occlusion, which significantly weaken structural boundaries and local texture cues. This observation suggests that, although the proposed model is more robust to viewpoint and appearance variations, its performance can still degrade when the scene structure is heavily obscured or when discriminative regional details are severely corrupted.

### 4.4. Ablation Study

#### 4.4.1. Effectiveness of Main Components

We conduct ablation studies to evaluate the contribution of each component in the proposed method. Specifically, we perform a componentwise analysis by separately adding the Multi-Scale Spatial Pyramid Attention (MSPA) module and the Coordinate Attention (CA) module to the CricaVPR baseline. All variants are trained on GSV-Cities under the same settings described in Section 4.2. The results on three standard benchmark datasets and three more challenging datasets are reported in Table 4 and Table 5, respectively.

As shown in Table 4, both MSPA and CA improve the baseline on the standard benchmarks. Compared with CricaVPR, adding MSPA increases R@1 by 0.3, 0.1, and 0.2 percentage points on Pitts250k-test, Tokyo 24/7, and Eynsham, respectively. Similarly, the addition of CA improves R@1 by 0.4, 0.4, and 0.1 percentage points on the same datasets. When both modules are used together, MscaVPR achieves the best overall performance, with increases in R@1 by 0.6, 0.8, and 0.3 percentage points over the baseline. These results indicate that MSPA and CA are beneficial and provide complementary improvements on conventional VPR benchmarks.

The results on the more challenging datasets in Table 5 further reveal the distinct roles of the two modules. MSPA alone yields clear gains on AmsterTime and SVOX-Night, improving R@1 by 0.8 and 1.7 percentage points, respectively, suggesting that multi-scale feature aggregation is particularly effective under long-term appearance changes and severe nighttime conditions. However, MSPA alone leads to a slight decrease on SVOX-Sun, indicating that it may be insufficient to handle complex weather and illumination variations without additional spatial refinement. In contrast, CA alone shows less consistent improvements than on the standard benchmarks and even causes a slight performance drop on AmsterTime, implying that coordinate attention is more sensitive to severe domain gaps when multi-scale contextual information is limited. Although the complete MscaVPR model shows a slightly lower R@5 than the single-module variants on SVOX-Night, it consistently provides the most accurate top one retrieval results on the three challenging datasets. This suggests that combining the two modules makes the top-ranked prediction more discriminative but may also slightly concentrate the similarity distribution on the most confident match, thereby reducing the diversity of the top retrieved candidates. These results also verify the complementarity between MSPA and CA: MSPA improves robustness to large appearance changes, while CA further enhances discriminative spatial cues, leading to more reliable place recognition under severe illumination, weather, and cross-domain variations.

**Figure 8:**
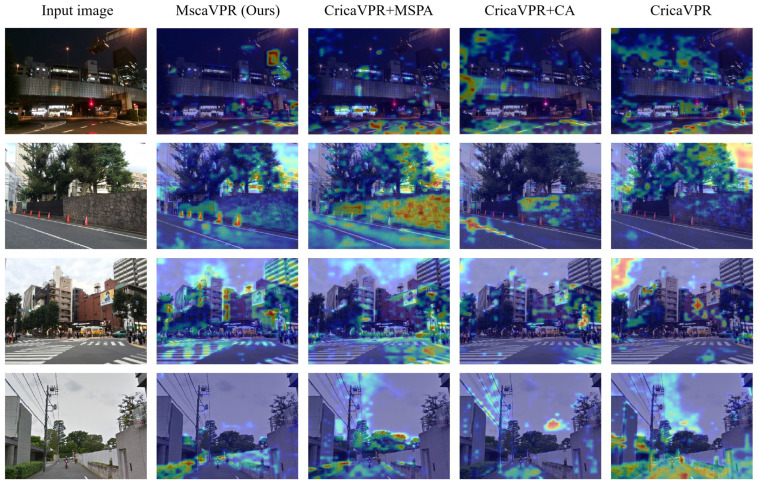
Grad-CAM visualizations of the output features for different model variants.

To further analyze the behavior of different model variants, we employ Grad-CAM [42] to visualize the output features of each model and generate the corresponding attention heatmaps. As shown in Figure 8, the baseline CricaVPR exhibits relatively diffuse attention, with considerable responses on less informative background regions such as the sky and road surfaces. After MSPA is integrated, the attention becomes more concentrated on building structures and local spatial layouts, while redundant background responses are reduced, indicating that multi-scale feature aggregation helps capture place-relevant cues more effectively. The CA module further refines the spatial distribution of attention, producing more localized responses on building edges, facade structures, and other semantically important regions. When the two modules are combined, MscaVPR shows the most focused and discriminative attention patterns, emphasizing structurally distinctive regions while further suppressing background interference. These observations are consistent with the quantitative improvements reported in Table 4 and Table 5.

#### 4.4.2. Sensitivity Analysis of the Azimuth Loss Weight

To evaluate the impact of azimuth-aware loss weight γ, we conduct a sensitivity study on three representative datasets, i.e., Pitts250k-test, MSLS-val, and AmsterTime, with γ∈{0.1,0.3,0.5,0.7}. The results are reported in Table 6. On Pitts250k-test, the performance varies only marginally across different γ values and shows a trend generally consistent with that on the more challenging AmsterTime dataset, with the best result achieved at γ=0.3. On MSLS-val, the best performance is achieved at γ=0.5 and is slightly better than the performance at γ=0.3. These results suggest that a moderate azimuth loss weight is generally beneficial, although the optimal value may vary slightly across datasets. When γ is too small, the orientation supervision may be insufficient to provide effective guidance, while excessively large values may overemphasize azimuth consistency and weaken the main objective of the retrieval. Therefore, considering both effectiveness and stability, we adopt γ=0.3 in all other experiments.

#### 4.4.3. Effectiveness of the Azimuth-Guided Training Strategy

We further evaluate the effect of the proposed azimuth-guided training strategy. Specifically, both CricaVPR and MscaVPR are trained under two settings: (1) a baseline setting using random sample selection with the standard multi-similarity (MS) loss, and (2) the proposed setting using azimuth-guided positive-pair selection together with the hybrid loss described in Section 3.5. All models are trained on GSV-Cities under the same hyperparameter settings as those in Section 4.2. The results on three standard benchmark datasets and three more challenging datasets are summarized in Table 7 and Table 8, respectively.

As shown in Table 7, the azimuth-guided training strategy brings limited changes to CricaVPR on Pitts250k-test and Eynsham but yields a clearer improvement on Tokyo 24/7, where R@1 increases by 0.6 percentage points, although R@5 decreases slightly. This suggests that azimuth guidance makes the top-ranked prediction more discriminative under richer viewpoint variations while slightly reducing the diversity of the top retrieved candidates. In contrast, for MscaVPR, the proposed strategy consistently improves performance on all three standard benchmark datasets, indicating that it is more effective when combined with stronger feature representations.

The gains become more evident on the challenging datasets in Table 8. For CricaVPR, azimuth-guided training improves R@1 by 0.1, 2.7, and 1.0 percentage points on AmsterTime, SVOX-Night, and SVOX-Sun, respectively. For MscaVPR, the corresponding gains are 0.3, 1.3, and 0.5 percentage points. These results indicate that the proposed strategy is particularly beneficial under larger viewpoint and appearance variations, where orientation information provides more effective supervision for positive-pair selection. Overall, although the magnitude of the improvement varies across datasets, the consistent gains on the more challenging benchmarks confirm that azimuth-guided training enhances the robustness of place representation learning.

## 5. Conclusions

In this paper, we presented MscaVPR, a visual place recognition framework that combines multi-scale feature enhancement with azimuth-aware training for improved robustness to appearance and viewpoint variations. Extensive experiments on multiple benchmark datasets demonstrate that MscaVPR consistently improves retrieval performance over a strong baseline and exhibits stronger robustness under challenging real-world conditions, including lighting, weather, temporal, and viewpoint changes. These results verify that explicitly leveraging azimuth information is an effective way to enhance viewpoint invariance in modern VPR frameworks.

Despite these improvements, several limitations remain. First, the proposed azimuth-guided strategy uses azimuth as the primary indicator of viewpoint discrepancy, although other imaging factors, such as focal length, pitch angle, and camera height, may also significantly affect the appearance of the same place. Consequently, the current formulation may not fully reflect the true difficulty of positive pairs. Second, the street-view datasets used in this work, such as GSV-Cities, provide limited viewpoint diversity, and the azimuth gap between co-located samples is often relatively small. This limits the evaluation of model robustness under more extreme viewpoint changes. In future work, we plan to incorporate additional geometric and camera-related factors into the mining process and to explore datasets with broader viewpoint coverage, such as 360°panoramic imagery or multi-view drone data, for more challenging viewpoint-robust VPR evaluation.

## Figures and Tables

**Table 1:** Overview of the test datasets used in the experiments.

Dataset	Number		Variations
	Queries	Database	
Pitts250k-test	8280	83,952	viewpoint, lighting, season
Tokyo 24/7	315	75,984	day/night, lighting, viewpoint
Eynsham	23,935	23,935	viewpoint, grayscale
MSLS-val	740	18,874	viewpoint, lighting, season
AmsterTime	1231	1231	long-term, season
SVOX-Night	823	17,166	viewpoint, day/night, lighting
SVOX-Sun	854		viewpoint, weather, lighting

**Table 2:** Comparison to state-of-the-art methods on three benchmark datasets. The best is highlighted in **bold**, and the second best is underlined.

Method	Pitts250k-Test		Tokyo 24/7		Eynsham		MSLS-Val	
	R@1	R@5	R@1	R@5	R@1	R@5	R@1	R@5
SFRS [41]	90.4	96.3	80.6	89.5	72.3	83.5	49.2	61.5
CosPlace [27]	91.7	97.0	89.5	94.9	89.9	93.8	67.5	78.1
EigenPlaces [10]	93.5	97.8	89.8	94.9	90.5	94.3	73.6	82.1
CricaVPR [13]	97.2	99.4	93.7	96.5	91.5	94.8	87.8	94.1
ClusVPR [31]	92.1	96.8	87.4	91.5	89.3	92.7	82.4	87.9
BoQ [30]	94.8	98.0	90.4	95.3	91.6	94.9	**88.5**	**94.5**
MscaVPR (Ours)	**97.8** ± 0.2	**99.5** ± 0.1	**94.5** ± 0.3	**96.8** ± 0.1	**91.8** ± 0.2	**95.1** ± 0.1	88.4 ± 0.3	**94.5** ± 0.1

**Table 3:** Comparison to state-of-the-art methods on more challenging datasets. The best is highlighted in **bold**, and the second best is underlined.

Method	AmsterTime		SVOX-Night		SVOX-Sun	
	R@1	R@5	R@1	R@5	R@1	R@5
SFRS [41]	29.7	48.5	28.7	40.6	54.8	68.3
CosPlace [27]	46.4	67.4	51.6	68.8	75.9	88.3
EigenPlaces [10]	45.7	68.5	51.5	70.8	83.1	93.8
CricaVPR [13]	63.0	82.5	88.2	95.0	93.2	97.9
ClusVPR [31]	42.3	62.7	46.8	67.1	72.2	85.4
BoQ [30]	52.1	71.4	88.9	95.5	93.6	98.4
MscaVPR (Ours)	**65.1** ± 0.3	**83.6** ± 0.2	**90.1** ± 0.3	**96.2** ± 0.2	**95.1** ± 0.3	**98.9** ± 0.1

**Table 4:** Ablation results of different model variants on three standard benchmark datasets. The best results are highlighted in **bold**.

Ablated Versions	Pitts250k-Test		Tokyo 24/7		Eynsham	
	R@1	R@5	R@1	R@5	R@1	R@5
CricaVPR (Baseline)	97.2	99.4	93.7	96.5	91.5	94.8
CricaVPR + MSPA	97.5 ± 0.2	99.4 ± 0.1	93.8 ± 0.2	96.5 ± 0.1	91.7 ± 0.2	95.0 ± 0.1
CricaVPR + CA	97.6 ± 0.2	**99.5** ± 0.1	94.1 ± 0.2	96.2 ± 0.1	91.6 ± 0.2	94.9 ± 0.1
MscaVPR (Ours)	**97.8** ± 0.2	**99.5** ± 0.1	**94.5** ± 0.3	**96.8** ± 0.1	**91.8** ± 0.2	**95.1** ± 0.1

**Table 5:** Ablation results of different model variants on three challenging datasets. The best results are highlighted in **bold**.

Ablated Versions	AmsterTime		SVOX-Night		SVOX-Sun	
	R@1	R@5	R@1	R@5	R@1	R@5
CricaVPR (Baseline)	63.0	82.5	88.2	95.0	93.2	97.9
CricaVPR + MSPA	63.8 ± 0.2	82.6 ± 0.1	89.9 ± 0.3	96.3 ± 0.2	93.0 ± 0.2	97.7 ± 0.1
CricaVPR + CA	62.1 ± 0.3	81.7 ± 0.1	88.2 ± 0.3	**96.4** ± 0.2	93.4 ± 0.2	98.0 ± 0.1
MscaVPR (Ours)	**65.1** ± 0.3	**83.6** ± 0.2	**90.1** ± 0.3	96.2 ± 0.2	**95.1** ± 0.3	**98.9** ± 0.1

**Table 6:** Ablation results of different value of azimuth loss weight on three typical datasets. The best results are highlighted in **bold**.

Parameter	Pitts250k-Test		MSLS-Val		AmsterTime	
	R@1	R@5	R@1	R@5	R@1	R@5
γ=0.7	97.4 ± 0.3	99.4 ± 0.1	87.3 ± 0.2	93.5 ± 0.1	63.8 ± 0.3	83.0 ±0.2
γ=0.5	97.7 ± 0.2	99.4 ± 0.1	**88.6** ± 0.3	**94.7** ± 0.2	64.7 ± 0.2	83.3 ± 0.1
γ=0.3	**97.8** ± 0.2	**99.5** ± 0.1	88.4 ± 0.3	94.5 ± 0.1	**65.1** ± 0.3	**83.6** ± 0.2
γ=0.1	97.6 ± 0.2	99.3 ± 0.1	87.8 ± 0.2	93.9 ± 0.1	64.3 ± 0.3	82.7 ± 0.1

**Table 7:** Ablation results of different training strategies on three benchmark datasets. The best is highlighted in **bold**.

Model	Training Strategy	Pitts250k-Test		Tokyo 24/7		Eynsham	
		R@1	R@5	R@1	R@5	R@1	R@5
CricaVPR (Baseline)	(1)	97.2	99.4	93.7	96.5	91.5	94.8
	(2)	97.3 ± 0.2	**99.5** ± 0.1	94.3 ± 0.2	96.0 ± 0.1	91.5 ± 0.2	94.9 ± 0.1
MscaVPR (Ours)	(1)	97.5 ± 0.2	99.4 ± 0.1	94.3 ± 0.3	96.5 ± 0.1	91.7 ± 0.3	95.0 ± 0.1
	(2)	**97.8** ± 0.2	**99.5** ± 0.1	**94.5** ± 0.3	**96.8** ± 0.1	**91.8** ± 0.2	**95.1** ± 0.1

**Table 8:** Ablation results of different training strategy on more challenging datasets. The best is highlighted in **bold**.

Model	Training Strategy	AmsterTime		SVOX-Night		SVOX-Sun	
		R@1	R@5	R@1	R@5	R@1	R@5
CricaVPR (Baseline)	(1)	63.0	82.5	88.2	95.0	93.2	97.9
	(2)	63.1 ± 0.2	82.8 ± 0.1	**90.9** ± 0.3	**96.3** ± 0.1	94.2 ± 0.2	98.8 ± 0.1
MscaVPR (Ours)	(1)	64.7 ± 0.2	83.1 ± 0.1	88.8 ± 0.3	**96.3** ± 0.2	94.6 ± 0.2	98.6 ± 0.1
	(2)	**65.1** ± 0.3	**83.6** ± 0.2	90.1 ± 0.3	96.2 ± 0.2	**95.1** ± 0.3	**98.9** ± 0.1

## Data Availability

The GSV-Cities dataset can be downloaded from https://www.kaggle.com/datasets/amaralibey/gsv-cities; the Pitts250k and Tokyo 24/7 datasets can be downloaded from https://data.ciirc.cvut.cz/public/projects/2015netVLAD/; the AmsterTime dataset can be downloaded from https://github.com/seyrankhademi/AmsterTime; the other datasets can be downloaded from https://github.com/gmberton/VPR-datasets-downloader. All datasets accessed on 15 April 2026.

## References

[1] Arandjelović R., Gronat P., Torii A., Pajdla T., Sivic J. (2018). NetVLAD: CNN Architecture for Weakly Supervised Place Recognition. IEEE Trans. Pattern Anal. Mach. Intell..

[2] Lowry S., Sünderhauf N., Newman P., Leonard J.J., Cox D., Corke P., Milford M.J. (2016). Visual Place Recognition: A Survey. IEEE Trans. Robot..

[3] Masone C., Caputo B. (2021). A Survey on Deep Visual Place Recognition. IEEE Access.

[4] McManus C., Churchill W., Maddern W., Stewart A.D., Newman P. (2014). Shady Dealings: Robust, Long-Term Visual Localisation Using Illumination Invariance. Proceedings of the 2014 IEEE International Conference on Robotics and Automation (ICRA).

[5] Nie J., Feng J.M., Xue D., Pan F., Liu W., Hu J., Cheng S. (2024). A Training-Free, Lightweight Global Image Descriptor for Long-Term Visual Place Recognition Toward Autonomous Vehicles. IEEE Trans. Intell. Transp. Syst..

[6] Jia Q., Zhong Z., Yang A., Wu M., Mu W., Liu F., Li P. (2025). HiStory: Methodology and System for GIS-based Narrative. Trans. GIS.

[7] Wang J., Han J., Dong R., Kan J. (2024). BinVPR: Binary Neural Networks towards Real-Valued for Visual Place Recognition. Sensors.

[8] Wu M., Jia Q., Yang A., Zhong Z., Ma M., Chen L., Jing N. (2025). Visual Route Recognition in Urban Spaces: A Scalable Approach Using Open Street View Data. IEEE J. Sel. Top. Appl. Earth Obs. Remote Sens..

[9] Grainge O., Milford M., Bodala I., Ramchurn S.D., Ehsan S. (2025). Structured Pruning for Efficient Visual Place Recognition. IEEE Robot. Autom. Lett..

[10] Berton G., Trivigno G., Caputo B., Masone C. (2023). EigenPlaces: Training Viewpoint Robust Models for Visual Place Recognition. Proceedings of the IEEE/CVF International Conference on Computer Vision (ICCV), Paris, France, 1–6 October 2023.

[11] Radenovic F., Tolias G., Chum O. (2019). Fine-Tuning CNN Image Retrieval with No Human Annotation. IEEE Trans. Pattern Anal. Mach. Intell..

[12] Wang R., Shen Y., Zuo W., Zhou S., Zheng N. (2022). TransVPR: Transformer-Based Place Recognition with Multi-Level Attention Aggregation. Proceedings of the 2022 IEEE/CVF Conference on Computer Vision and Pattern Recognition (CVPR).

[13] Lu F., Lan X., Zhang L., Jiang D., Wang Y., Yuan C. (2024). CricaVPR: Cross-image Correlation-Aware Representation Learning for Visual Place Recognition. Proceedings of the 2024 IEEE/CVF Conference on Computer Vision and Pattern Recognition (CVPR).

[14] Yu Y., Zhang Y., Cheng Z., Song Z., Tang C. (2024). Multi-Scale Spatial Pyramid Attention Mechanism for Image Recognition: An Effective Approach. Eng. Appl. Artif. Intell..

[15] Hou Q., Zhou D., Feng J. (2021). Coordinate Attention for Efficient Mobile Network Design. Proceedings of the 2021 IEEE/CVF Conference on Computer Vision and Pattern Recognition (CVPR).

[16] Lowe D.G. (2004). Distinctive Image Features from Scale-Invariant Keypoints. Int. J. Comput. Vis..

[17] Dalal N., Triggs B. (2005). Histograms of oriented gradients for human detection. Proceedings of the 2005 IEEE Computer Society Conference on Computer Vision and Pattern Recognition (CVPR’05).

[18] Bay H., Tuytelaars T., Van Gool L., Leonardis A., Bischof H., Pinz A. (2006). SURF: Speeded up Robust Features. Computer Vision—ECCV 2006.

[19] Rublee E., Rabaud V., Konolige K., Bradski G. (2011). ORB: An Efficient Alternative to SIFT or SURF. Proceedings of the 2011 International Conference on Computer Vision.

[20] Galvez-López D., Tardos J.D. (2012). Bags of Binary Words for Fast Place Recognition in Image Sequences. IEEE Trans. Robot..

[21] Perronnin F., Sánchez J., Mensink T., Daniilidis K., Maragos P., Paragios N. (2010). Improving the Fisher Kernel for Large-Scale Image Classification. Computer Vision—ECCV 2010.

[22] Jégou H., Douze M., Schmid C., Pérez P. (2010). Aggregating Local Descriptors into a Compact Image Representation. Proceedings of the 2010 IEEE Computer Society Conference on Computer Vision and Pattern Recognition.

[23] Simonyan K., Zisserman A. (2014). Very Deep Convolutional Networks for Large-Scale Image Recognition. arXiv.

[24] He K., Zhang X., Ren S., Sun J. (2016). Deep Residual Learning for Image Recognition. Proceedings of the 2016 IEEE Conference on Computer Vision and Pattern Recognition (CVPR).

[25] Hausler S., Garg S., Xu M., Milford M., Fischer T. (2021). Patch-NetVLAD: Multi-Scale Fusion of Locally-Global Descriptors for Place Recognition. Proceedings of the 2021 IEEE/CVF Conference on Computer Vision and Pattern Recognition (CVPR).

[26] Ali-bey A., Chaib-draa B., Giguère P. (2023). MixVPR: Feature Mixing for Visual Place Recognition. Proceedings of the IEEE/CVF Winter Conference on Applications of Computer Vision.

[27] Berton G., Masone C., Caputo B. (2022). Rethinking Visual Geo-localization for Large-Scale Applications. Proceedings of the 2022 IEEE/CVF Conference on Computer Vision and Pattern Recognition (CVPR).

[28] Berton G.M., Masone C., Paolicelli V., Caputo B. (2021). Viewpoint Invariant Dense Matching for Visual Geolocalization. 2021 IEEE/CVF International Conference on Computer Vision (ICCV).

[29] Gkelios S., Boutalis Y., Chatzichristofis S.A. (2021). Investigating the Vision Transformer Model for Image Retrieval Tasks. 2021 17th International Conference on Distributed Computing in Sensor Systems (DCOSS).

[30] Ali-bey A., Chaib-draa B., Giguère P. (2024). BoQ: A Place Is Worth a Bag of Learnable Queries. Proceedings of the 2024 IEEE/CVF Conference on Computer Vision and Pattern Recognition (CVPR).

[31] Xu Y., Shamsolmoali P., Zareapoor M., Yang J. (2025). ClusVPR: Efficient Visual Place Recognition with Clustering-Based Weighted Transformer. IEEE Trans. Artif. Intell..

[32] Tzachor I., Lerner B., Levy M., Green M., Berkovitz Shalev T., Habib G., Samuel D., Zailer N., Shimshi O., Darshan N., Yue Y., Garg A., Peng N., Sha F., Yu R. (2025). EffoVPR: Effective Foundation Model Utilization for Visual Place Recognition. Proceedings of the International Conference on Learning Representations.

[33] Oquab M., Darcet T., Moutakanni T., Vo H.V., Szafraniec M., Khalidov V., Fernandez P., Haziza D., Massa F., El-Nouby A. (2023). DINOv2: Learning Robust Visual Features without Supervision. arXiv.

[34] Wang X., Han X., Huang W., Dong D., Scott M.R. (2019). Multi-Similarity Loss with General Pair Weighting for Deep Metric Learning. Proceedings of the 2019 IEEE/CVF Conference on Computer Vision and Pattern Recognition (CVPR).

[35] Ali-bey A., Chaib-draa B., Giguère P. (2022). GSV-cities: Toward Appropriate Supervised Visual Place Recognition. Neurocomputing.

[36] Torii A., Sivic J., Okutomi M., Pajdla T. (2015). Visual Place Recognition with Repetitive Structures. IEEE Trans. Pattern Anal. Mach. Intell..

[37] Torii A., Arandjelović R., Sivic J., Okutomi M., Pajdla T. (2015). 24/7 Place Recognition by View Synthesis. Proceedings of the 2015 IEEE Conference on Computer Vision and Pattern Recognition (CVPR).

[38] Cummins M., Newman P. (2011). Appearance-Only SLAM at Large Scale with FAB-MAP 2.0. Int. J. Robot. Res..

[39] Warburg F., Hauberg S., López-Antequera M., Gargallo P., Kuang Y., Civera J. (2020). Mapillary Street-Level Sequences: A Dataset for Lifelong Place Recognition. Proceedings of the 2020 IEEE/CVF Conference on Computer Vision and Pattern Recognition (CVPR).

[40] Yildiz B., Khademi S., Siebes R.M., Van Gemert J. (2022). AmsterTime: A Visual Place Recognition Benchmark Dataset for Severe Domain Shift. Proceedings of the 2022 26th International Conference on Pattern Recognition (ICPR).

[41] Ge Y., Wang H., Zhu F., Zhao R., Li H. (2020). Self-supervising Fine-Grained Region Similarities for Large-Scale Image Localization. Proceedings of the 2020 European Conference on Computer Vision (ECCV).

[42] Selvaraju R.R., Cogswell M., Das A., Vedantam R., Parikh D., Batra D. (2017). Grad-CAM: Visual Explanations from Deep Networks via Gradient-Based Localization. Proceedings of the 2017 IEEE International Conference on Computer Vision (ICCV).

